# Career Adaptability in Special Educational Needs Populations: A Systematic Review of the Empirical Evidence and Emerging Research Directions

**DOI:** 10.3390/bs15070927

**Published:** 2025-07-09

**Authors:** Cheng Li, Lan Yang, Kuen Fung Sin, Fengzhan Gao, Alessandra Romano

**Affiliations:** 1 Institute of Special Needs and Inclusive Education (ISNIE), The Education University of Hong Kong, Hong Kong, China; lic@eduhk.hk (C.L.); kfsin@eduhk.hk (K.F.S.); fzgao@eduhk.hk (F.G.); 2 D1-1/F-44, Department of Curriculum and Instruction, The Education University of Hong Kong, Hong Kong, China; 3 Analytics\Assessment Research Centre (ARC), The Education University of Hong Kong, Hong Kong, China; 4 Department of Special Education and Counselling, The Education University of Hong Kong, Hong Kong, China; 5 Department of Social, Political and Cognitive Sciences, University of Siena, 53100 Siena, Italy; alessandra.romano2@unisi.it

**Keywords:** career adaptability, special educational needs, systematic review, career development, inclusive education

## Abstract

Despite robust evidence linking career adaptability (CA) to positive vocational and psychosocial outcomes in general populations, research on the CA among individuals with special educational needs (SEN) remains limited. Prior reviews have largely overlooked the distinct challenges faced by SEN populations. To address this gap, we conducted a systematic review across five major databases, yielding an initial pool of 81 studies. Following rigorous screening, only eight quantitative studies met the inclusion criteria, reflecting the early stage of the research in this area. The included studies span diverse SEN groups, including individuals with visual impairments, intellectual disabilities, and mental health conditions. CA was consistently found to be associated with adaptive outcomes such as self-esteem, self-efficacy, hope, and career satisfaction. However, the literature is characterized by methodological limitations, notably the predominance of cross-sectional designs, the underrepresentation of neurodevelopmental conditions (e.g., ASD, ADHD), and a lack of cross-cultural perspectives and standardized instruments specifically adapted to SEN learners. Future studies should focus on the need for longitudinal and mixed-method designs, contextually cross-cultural research, and inclusive measurement tools. Furthermore, exploring the ecological and emotional predictors of CA; expanding to underrepresented SEN subgroups; and evaluating diverse interventions beyond mentoring are essential to informing tailored educational and vocational support for individuals with SEN.

## 1. Introduction

Career adaptability is a core construct within career construction theory (CCT), which provides a robust framework for understanding how individuals prepare for and manage career transitions. As defined by Savickas (2005), career adaptability is “a psychosocial construct that denotes an individual’s readiness and resources for coping with current and imminent vocational development tasks, occupational transitions, and personal traumas” (p. 51). Grounded in career construction theory, it comprises four interrelated dimensions—concern, control, curiosity, and confidence (Savickas & Porfeli, 2011)—that together describe how individuals navigate career-related demands. Concern reflects future orientation and planning; control involves taking ownership of career decisions; curiosity entails exploring vocational possibilities; and confidence refers to self-efficacy in overcoming challenges. In the context of students with special educational needs (SEN), these dimensions may be expressed through setting post-school goals (concern), engaging in Individualized Education Plan (IEP) decision-making (control), exploring work-based learning opportunities (curiosity), and persisting through training challenges (confidence). In recent decades, extensive research has demonstrated that higher levels of career adaptability are associated with significant positive outcomes, including enhanced self-esteem, hope, life satisfaction, and overall well-being (Johnston, 2018). Meta-analyses by Rudolph et al. (2017a, 2017b) have provided compelling evidence that career adaptability is positively correlated with various career-related constructs—such as career planning, exploration, self-efficacy, and decision-making—as well as with favorable career outcomes such as job satisfaction, employability, income, and overall life satisfaction. Furthermore, Johnston’s (2018) comprehensive review, which encompassed 116 publications, highlighted the dual role of career adaptability as both a resource and a catalyst for adaptive vocational responses. Johnston’s work also revealed significant heterogeneity in the measurement of career adaptability, with identified instruments varying from 12 to 55 items, thus underscoring the ongoing need for methodological refinement and consistency.

Despite this burgeoning body of literature, research on the career adaptability among individuals with special educational needs (SEN) or disabilities remains markedly underexplored in the international educational literature. Given that individuals with disabilities often face additional barriers in the workforce compared to those for their typically developing peers (Lindsay et al., 2015; Lindstrom et al., 2013), an enhanced understanding of their career adaptability is critical for informing interventions tailored to their unique challenges and resource profiles (Ferrari et al., 2017; Santilli et al., 2014). In light of these considerations, it is imperative to adopt a structured, evidence-based approach to synthesizing and critically evaluating the existing literature. To this end, our research employs a systematic review of primary studies focused specifically on SEN populations.

### Career Adaptability in Individuals with Disabilities

Previous studies have shown that it is often difficult for students with disabilities to seek for jobs or enter higher tertiary institutes after graduating from secondary school (Sin & Yang, 2018; Horvath-Rose et al., 2004). According to the interviews with parents of students with disabilities in Sin and Yang’s (2018) study, most parents have high expectations that their children can continue learning in higher education, no matter what major. However, the opportunities and channels for helping students with disabilities with the post-school transition are limited, and parents often find themselves helpless or hopeless in supporting their children in this transition (Sin & Yang, 2018). Therefore, more support focused on career training for students with disabilities is warranted in order to help these students develop their career adaptability based on their unique disability experiences.

In recent years, a greater number of studies have tried to understand the career adaptability of those with disabilities and to demonstrate the importance of delivering career adaptability skill training to this group of people, such as training in problem-solving skills, time management competence, emotion control skills, and communication skills (Lindstrom et al., 2013; Yang et al., 2015). Although some studies have investigated the relationship between career adaptability and positive psychological constructs (Santilli et al., 2014; Yang et al., 2023; Yuen & Chan, 2024), the number of such studies is scarce. Moreover, currently, no reviews or meta-analyses on the career adaptability of individuals with disabilities have yet been conducted. It is conceived that a review on the career adaptability in individuals with disabilities would attract more attention from research and practice to these groups of people with disabilities and provide possible directions for future studies.

## 2. Study Identification and Selection Process

**Figure 1:**
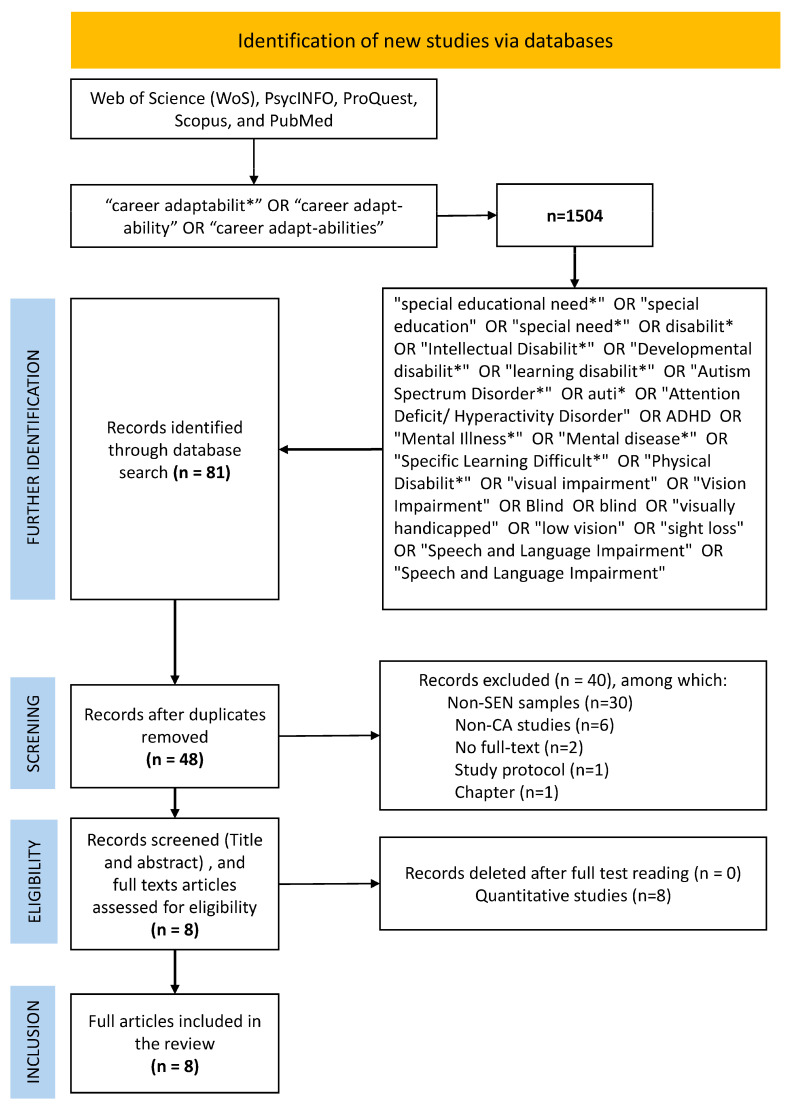
PRISMA 2020 flow diagram of study identification and selection. Note: The asterisk (*) symbol is used as a wildcard, specifically for truncation.

This systematic review was conducted in strict accordance with the PRISMA 2020 (Preferred Reporting Items for Systematic Reviews and Meta-Analyses) guidelines (Page et al., 2021). All procedural steps—identification, screening, eligibility assessment, and inclusion—were transparently documented and reported in alignment with the PRISMA 2020 framework. The entire process is visually represented in Figure 1, which adheres to the standard PRISMA 2020 flow diagram format, and a complete checklist is provided in Table A2 to ensure reporting compliance at each item level.

A comprehensive and systematic search strategy was executed across five major academic databases (i.e., Web of Science, PsycINFO, ProQuest, Scopus, and PubMed) to identify empirical research situated at the intersection of career adaptability (“career adaptabilit*” OR “career adapt-ability” OR “career adapt-abilities”) and special educational needs (SEN) (“special educational need*” OR “special education” OR “special need*” OR disabilit* OR “Intellectual Disabilit*” OR “Developmental disabilit*” OR “learning disabilit*” OR “Autism Spectrum Disorder*” OR auti* OR “Attention Deficit/Hyperactivity Disorder” OR ADHD OR “Mental Illness*” OR “Mental disease*” OR “Specific Learning Difficult*” OR “Physical Disabilit*” OR “visual impairment” OR “Vision Impairment” OR Blind OR blind OR “visually handicapped” OR “low vision” OR “sight loss” OR “Speech and Language Impairment” OR “Speech and Language Impairment”). The specific search terms, logic, and rationale are systematically detailed in Table 1 to ensure replicability and coverage.

This initial search yielded 1504 records. The literature search did not apply any geographic restrictions; however, only English-language, peer-reviewed publications were included to ensure consistency and accessibility. After applying inclusion filters and removing irrelevant content based on database search constraints, 81 records were further identified through a systematic database search (see Figure 1). After the removal of 33 duplicates, 48 records remained for screening. During the title and abstract screening phase, 40 records were excluded, including 31 studies that did not involve participants with special educational needs (SEN), 4 studies that were unrelated to the construct of career adaptability (CA), 2 studies that did not provide full-text access, 1 study that was a study protocol, and 2 studies that were book chapters rather than peer-reviewed journal articles. For transparency, the demographic details of all 48 screened records are provided in Appendix A (Table A1) for readers who wish to examine the full scope of the screening process.

### Researcher Reflexivity and Positionality

This review was conducted by five researchers with expertise in special educational needs (SEN) education. Four are based in Hong Kong, drawing on their local educational experience, while one is based in Italy, offering an international comparative perspective. Our professional and cultural backgrounds informed how we framed the research questions, interpreted the findings, and selected relevant studies.

To reduce potential bias, we engaged in collaborative discussions throughout the review process, particularly during the screening and analysis, to ensure diverse viewpoints were considered. By acknowledging our positionality and applying reflexive practices, we aimed to enhance the transparency and trustworthiness of our interpretations and findings.

A comprehensive search was conducted across five major academic databases—Web of Science, PsycINFO, ProQuest, Scopus, and PubMed—using a carefully constructed set of search terms to capture the intersection of career adaptability and special educational needs (SEN). We present the details on each searching term via Table 1.

## 3. Results

Ultimately, eight quantitative studies were included in the final synthesis. This rigorous selection process underscores the limited but emerging empirical base addressing career adaptability (CA) in special educational needs (SEN) populations and highlights the methodological transparency of this review. These studies provide critical insights into the measurement, validation, and potential intervention strategies aimed at enhancing CA among individuals facing diverse vocational challenges. The following paragraphs elaborate on the key features and findings of these studies.

Salimi et al. (2023) conducted a cross-sectional study with 319 Iranian adults with visual impairments, revealing a positive association among career adaptivity, CA, and career satisfaction. These findings imply that embedding CA strategies within career training programs could potentially enhance the career satisfaction in this population. In a separate study, Yang et al. (2023) focused on 204 SEN students in Hong Kong to validate the psychometric properties of the Career Adaptation Scale–Short Form (CAAS-SF). Their findings supported a four-factor model of CA and identified a positive relationship between CA and self-esteem, confirming the scale’s reliability and potential utility for targeted career guidance interventions.

Yuen and Chan (2024) adopted a longitudinal design, assessing 345 SEN students alongside 237 matched participants in Hong Kong across an 18-month interval. Their analyses demonstrated that a robust sense of meaning in life significantly predicted both CA and career self-efficacy, emphasizing the importance of internal motivational resources in adaptive career behaviors among SEN populations. Similarly, Stevenson et al. (2021) employed a cross-sectional methodology involving 85 individuals with serious mental illnesses in the United States. Their findings indicated that while work hope significantly predicted job search intensity, an unexpected negative correlation emerged between CA and job search intensity among the employed participants. Additionally, the psychometric validity of the work hope and CA scales was limited in this sample, highlighting the need for further research to tailor CA interventions to individuals with mental health challenges.

Tokar and Kaut (2018) evaluated 320 workers diagnosed with Chiari malformations in the United States, finding that CA partially mediated the relationship between economic constraints and access to decent work. This suggests that CA may serve as a protective factor against adverse economic conditions and that further exploration into the role of mental health in vocational outcomes is warranted. In Italy, Santilli et al. (2014) conducted a cross-sectional study involving 120 adults with intellectual disability, in which hope was found to partially mediate the relationship between career adaptability and life satisfaction. These results imply that positive constructs like hope and CA can help improve life satisfaction and that career training programs for individuals with disabilities could benefit from focusing on these elements.

Two intervention studies by O’Mally and Antonelli (2016) and Antonelli et al. (2018) examined the effectiveness of mentoring programs among legally blind college students in the United States. O’Mally and Antonelli (2016) paired 26 legally blind students with employed mentors and observed significant improvements in their job-seeking assertiveness, career self-efficacy, and CA. Antonelli et al. (2018), using the same sample, replicated these findings and demonstrated that the mentoring program effectively enhanced both efficient job searching and CA. Collectively, these intervention studies suggest that structured mentoring is a viable approach to bolstering vocational competencies in SEN populations.

Together, these eight studies not only illustrate what has been accomplished in terms of validating CA measures and elucidating CA’s relationship with key psychological constructs but also raise critical questions regarding future directions. What innovative interventions can further sustain and enhance CA among SEN students over time? How could these findings be integrated into broader career development frameworks to promote equitable and effective vocational outcomes? Addressing these questions is essential for advancing the research and informing policy and practice in the field of career development for individuals with special educational needs.

### 3.1. Participants’ Characteristics

The review included diverse participant groups with SEN as follows: one study investigated CA among 319 adults with visual impairments (Salimi et al., 2023), while two studies utilized a shared sample of 77 legally blind college students paired with employed mentors (Antonelli et al., 2018; O’Mally & Antonelli, 2016). One study focused on 120 adults with mild intellectual disabilities (Santilli et al., 2014). One study examined 320 workers diagnosed with Chiari malformation type 1 (Tokar & Kaut, 2018), and another assessed 85 individuals with one or more mental health conditions (Stevenson et al., 2021). Additionally, two studies targeted secondary school students across at least seven types of SEN, yielding a combined sample of 549 participants (Yang et al., 2023; Yuen & Chan, 2024).

### 3.2. The Geographical Distribution

The studies were geographically diverse: four were conducted in the United States (Antonelli et al., 2018; O’Mally & Antonelli, 2016; Stevenson et al., 2021; Tokar & Kaut, 2018), two in Hong Kong (Yang et al., 2023; Yuen & Chan, 2024), one in Iran (Salimi et al., 2023), and one in Italy (Santilli et al., 2014). This distribution is noteworthy given that the majority of the research originates from developed regions, where structured support systems and policies for special educational needs (SEN) have been more extensively established and studied. It is also important to consider that the observed geographical concentration may partly reflect the selective inclusion of English-language publications in the analysis. Despite these advancements, significant unemployment remains among working-age individuals with disabilities, being estimated at 50% to 70% in industrialized areas and up to 90% in developing regions (*Disability and Employment|United Nations Enable*, n.d.). This juxtaposition reveals a critical research gap: even in well-resourced contexts, the investigation into career adaptability among SEN populations is limited and lacks depth, underscoring the urgent need for more comprehensive studies in this area.

### 3.3. The Research Contexts

This review showed that most of the studies on career adaptability have been conducted in developed settings, specifically the United States, Italy, and Hong Kong. In these contexts, significant advances in special educational needs (SEN) provisions have taken place over the past two to three decades—for example, the United States has offered free and appropriate public education to children with disabilities since the Individuals with Disabilities Education Act (IDEA) of 1990; Italy has implemented full school inclusion for students with disabilities since Law 104/1992 (Law 104/1992, 1992); and Hong Kong has seen ongoing enhancements in SEN policy and resource allocation since the late 1990s (Dai, 2024). These developments reflect sustained policy commitments and infrastructure growth in SEN education. Nevertheless, despite these advancements, the employment rates among individuals with disabilities remain concerningly low. According to the United Nations, 80% to 90% of working-age persons with disabilities in developing countries are unemployed, while in industrialized countries, the unemployment rate ranges from 50% to 70%. Moreover, across all regions, persons with disabilities are consistently two to three times more likely to be unemployed than their non-disabled peers (*Disability and Employment|United Nations Enable*, n.d.). This disparity highlights the urgent need for in-depth investigation of CA in SEN populations, even in settings where the overall support for SEN is relatively well developed.

### 3.4. The Research Design

The eight studies primarily employed quantitative methodologies, with five adopting cross-sectional designs and three incorporating longitudinal frameworks. Among the longitudinal studies, Yuen and Chan (2024) uniquely explored the long-term associations between CA, career self-efficacy, and social connectedness. The other two longitudinal investigations, conducted by O’Mally and Antonelli (2016) and Antonelli et al. (2018), focused on the efficacy of mentoring programs in enhancing CA and job search behaviors among legally blind college students. Collectively, these designs indicate an overall scarcity in research exploring the interplay between CA and other psychological constructs within SEN samples, thereby inviting further longitudinal investigations.

### 3.5. Measurement of Career Adaptability

All studies assessed CA using standardized self-report instruments, adopting one of three approaches. First, the original 24-item version of the Career Adapt-Ability Scale (CAAS; Savickas & Porfeli, 2011) was used in the studies by Salimi et al. (2023), Stevenson et al. (2021), and Santilli et al. (2014). Second, the shortened 12-item CAAS-SF (Maggiori et al., 2017) was employed by Yang et al. (2023), Yuen and Chan (2024), and Tokar and Kaut (2018). And third, the career adaptability scale (CAS; Rottinghaus et al., 2017) was utilized in both O’Mally and Antonelli (2016) and Antonelli et al. (2018). The instruments demonstrated acceptable reliability, with the Cronbach’s alpha values ranging from 0.71 to 0.95 across studies (Salimi et al., 2023; Santilli et al., 2014; Stevenson et al., 2021; Tokar & Kaut, 2018; Yang et al., 2023). Moreover, Yuen and Chan (2024) reported McDonald’s omega coefficients for the four subscales, which ranged from 0.78 to 0.80, further substantiating the internal consistency of the measures.

Table 2 summarizes the diverse methodological approaches, measurement strategies, and thematic foci employed in these studies. Together, they have contributed to establishing a foundation for understanding how CA functions in SEN populations, informing both current practice and future research directions aimed at enhancing vocational outcomes for these individuals with SEN.

## 4. Discussion

The literature on career adaptability (CA), as synthesized in this review focused on special educational needs (SEN) populations, reveals both promising findings and notable limitations. Previous reviews such as those by Rudolph et al. (2017a, 2017b) and Johnston (2018) have robustly established the multidimensional nature of CA and its significant positive associations with various career and well-being outcomes. However, these reviews predominantly rely on data from typically developing samples.

Our systematic search identified eight quantitative studies that have mapped the landscape of CA research within SEN populations, ranging from secondary school students to adults with various disabilities. The eight studies reveal that consistent with findings in the general population, CA in individuals with SEN is positively linked to beneficial career and life outcomes such as self-esteem, hope, career self-efficacy, and career satisfaction (Salimi et al., 2023; Yang et al., 2023; Yuen & Chan, 2024). Nevertheless, the nuances in these associations underscore the importance of adapting established theoretical models, such as career construction theory and career adaptation theory, to reflect the specific experiences of SEN students and adults. For example, although Salimi et al. (2023) demonstrate a positive relationship between career adaptivity, CA, and career satisfaction among adults with visual impairments, only a limited number of studies have explored how the interplay among the dimensions of CA manifests across different types of SEN. Moreover, intervention studies (Antonelli et al., 2018; O’Mally & Antonelli, 2016) provide preliminary evidence that mentoring programs can enhance CA among SEN participants, suggesting practical avenues for vocational interventions; however, these findings await further replication and contextual expansion.

**Figure 2:**
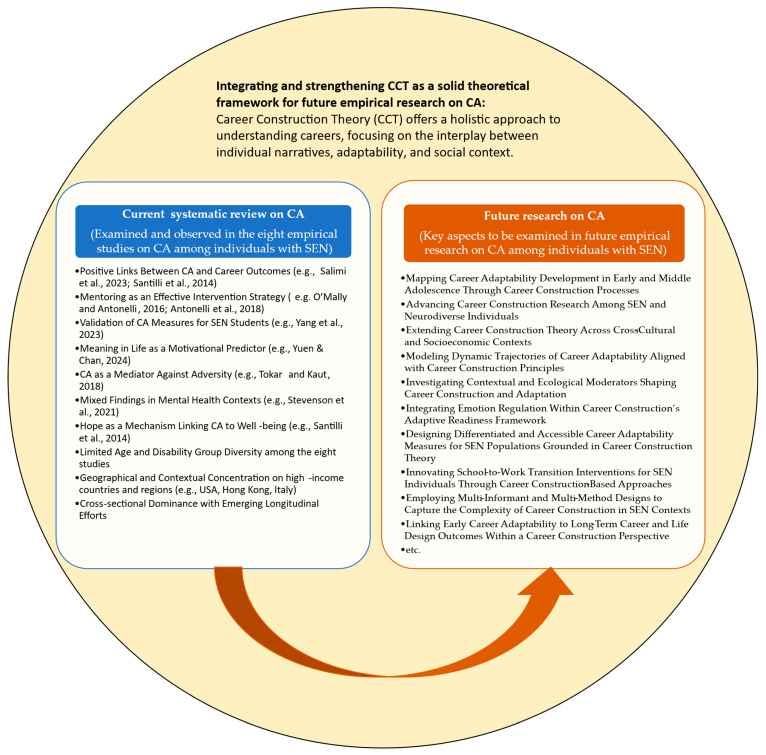
An overview of what has been covered and what can be pursued next in future research by explicitly integrating and strengthening CCT to advance CA research. (Antonelli et al., 2018; O’Mally & Antonelli, 2016; Salimi et al., 2023; Santilli et al., 2014; Stevenson et al., 2021; Tokar & Kaut, 2018; Yang et al., 2023; Yuen & Chan, 2024).

This study is not without limitations. The major limitation would be the limited scope on individuals with SEN resulting in a small number of articles reviewed. While the inclusion of only eight studies may appear limited in numerical terms, this narrow scope is a direct reflection of the nascent state of the empirical research situated at the intersection of career adaptability and special educational needs. The systematic review was conducted using a comprehensive, multi-database search strategy guided by the PRISMA standards, applying clearly defined inclusion criteria to ensure conceptual and methodological relevance. The low yield is not indicative of inadequate search procedures but rather highlights a substantive gap in the literature. This gap underscores the importance of the review itself: to formally map and assess the sparse yet emerging body of evidence in an area that remains underrepresented despite its high theoretical and applied significance. By rigorously analyzing the design features, populations, theoretical frameworks, and measurement strategies used in the existing literature, this review provides a focused, evidence-informed foundation for future research to advance both the scholarly and applied discourse on inclusive career development. The limited number of studies is thus not a weakness but a critical finding in itself, pointing to an urgent future research agenda (see Figure 2) and justifying the need for increased attention on equity-focused career adaptability research. Building upon these initial insights, the following section outlines key future research directions derived from the current evidence base.

### Informing Future Research Directions from the Current Insights

The systematic review of these eight studies offers an important initial mapping of how career adaptability has been conceptualized, measured, and linked to career outcomes among individuals with special educational needs. These studies collectively affirm that CA is a meaningful psychological resource for promoting career development, aligning with the core propositions of career construction theory (Savickas, 2005, 2013).

Most studies, however, have employed cross-sectional designs, which constrain the ability to discern causal relationships between CA and career outcomes over time. In alignment with career construction theory, future research should prioritize modeling the dynamic trajectories of career adaptability through longitudinal designs, capturing the evolving construction of vocational identity among individuals with SEN. While a few longitudinal studies (e.g., Yuen & Chan, 2024) have begun to explore these dynamics, the temporal evolution of CA among individuals with SEN remains largely unexplored. Moreover, the SEN samples included in these studies are not sufficiently diverse; many focus on specific groups—such as individuals with visual impairments or intellectual disabilities—leaving other SEN categories (e.g., those with autism spectrum disorders, learning disabilities, or physical impairments) underrepresented. Advancing the career construction research among a broader range of individuals with SEN and neurodiverse individuals is essential to building a more comprehensive and ecologically valid understanding of adaptability across heterogeneous developmental pathways. Future studies should also consider mapping the development of career adaptability specifically during early and middle adolescence, a critical period for the construction of self-concept and future orientation, consistent with CCT’s lifespan perspective. This concentration currently hinders the development of a comprehensive model that accurately reflects the heterogeneous nature of SEN populations.

Measurement issues also pose a significant challenge. The studies reviewed uniformly employ instruments such as the Career Adapt-Ability Scale (CAAS), its short form (CAAS-SF), and the career adaptability scale (CAS). Although these tools have demonstrated acceptable reliability (Cronbach’s alpha values ranging from 0.71 to 0.95 and McDonald’s omega coefficients between 0.78 and 0.80), they were originally validated in samples of typically developing individuals and might not fully capture the multidimensional and context-specific aspects of CA in SEN populations. Given CCT’s emphasis on adaptability as a context-sensitive psychosocial resource, future research should focus on designing differentiated and accessible career adaptability measures tailored to SEN populations, ideally by extending and refining the conceptual foundation laid by the CAAS.

In addition, future studies should include participants across a broader spectrum of SEN, encompassing various cognitive, physical, and developmental challenges, as well as recruiting from different cultural and socioeconomic backgrounds. Extending career construction theory across cross-cultural and socioeconomic contexts, including low- and middle-income countries, is critical to enhancing the global relevance and equity of career adaptability research. This expansion would enhance the ecological validity of the research findings and inform the development of more tailored and equitable intervention strategies. A more precise delineation of the disability continuum in relation to career outcomes warrants further investigation, particularly to examine how varying degrees and types of disability influence individuals’ independence in workplace environments. Recent findings also suggest that disability severity shapes coping strategies and perceptions of professional isolation, which in turn affect career outcomes differently across impairment levels (Lyons et al., 2023). Furthermore, intervention studies need to be scaled up and systematically evaluated. While the preliminary evidence from mentoring programs (e.g., Antonelli et al., 2018; O’Mally & Antonelli, 2016) indicates that targeted interventions may effectively enhance CA among SEN individuals, future efforts should innovate school-to-work transition interventions grounded in career construction principles, focusing not only on skill acquisition but also on fostering adaptive readiness, narrative identity, and self-authorship. Employing multi-informant and multi-method research designs will also be valuable for capturing the complex, co-constructed nature of career adaptability processes in SEN contexts. Additionally, investigating contextual and ecological moderators, such as family, school, and community influences, will provide richer insights into how career adaptability is constructed and sustained across diverse environments. Integrating emotion regulation as part of adaptive readiness mechanisms could elucidate further how affective competencies interact with adaptability among individuals with SEN, offering an affectively enriched career construction framework.

In conclusion, while the findings from the studies reviewed offer encouraging support for the role of CA as an adaptation resource facilitating positive career outcomes within a career construction perspective, significant gaps remain. Future research should link early career adaptability development to long-term career and life design outcomes, capturing the full arc of vocational and psychosocial construction envisioned by CCT. Research efforts grounded in CCT and employing longitudinal, developmentally sensitive, culturally diverse, and methodologically robust approaches are essential to enrich our understanding of the CA in SEN populations. Such advances with further efforts will not only refine the theoretical models but also inform the development of evidence-based practices that can ultimately contribute to improving career development and vocational inclusion for individuals with special educational needs.

## Figures and Tables

**Table 1:** Elaboration of the search strategies for each SEN type in this systematic review as supplementary information to Figure 1.

Keywords Concerned in Our Current Review	Keywords Used by Us	Our Aims
1. Career Adaptability	“career adaptabilit*” OR “career adapt-ability” OR “career adapt-abilities”	To capture all relevant studies discussing career adaptability, including different spellings and suffixes.
2. Special Educational Needs (SEN)	“special educational need*” OR “special education” OR “special need*”	To include studies focusing on any aspect of special educational needs.
3. Disabilities (General)	“disabilit*”	To ensure the inclusion of studies mentioning any type of disabilities.
4. Intellectual and Developmental Disabilities	“Intellectual Disabilit*” OR “Developmental disabilit*” OR “learning disabilit*”	To cover studies focused on intellectual and developmental disabilities, as well as learning disabilities.
5. Autism Spectrum Disorders	“Autism Spectrum Disorder*” OR “auti*”	To capture studies related to autism spectrum disorders using various terminologies.
6. Attention Deficit/Hyperactivity Disorder	“Attention Deficit/Hyperactivity Disorder” OR “ADHD”	To ensure comprehensive coverage of studies related to ADHD, including both the full term and the acronym.
7. Mental Health Conditions	“Mental Illness*” OR “Mental disease*”	To include studies discussing various mental health conditions.
8. Specific Learning Difficulties	“Specific Learning Difficult*”	To capture studies focusing on specific learning difficulties, covering different terminologies used.
9. Physical Disabilities	“Physical Disabilit*”	To ensure the inclusion of studies related to physical disabilities, using a wildcard to capture various suffixes.
10. Visual Impairments	“visual impairment” OR “Vision Impairment” OR “Blind” OR “blind” OR “visually handicapped” OR “low vision” OR “sight loss”	To cover a range of visual impairments and ensure no relevant studies are missed due to variations in terminology.
11. Speech and Language Impairments	“Speech and Language Impairment”	To include studies focusing on speech and language impairments.

Note: SEN = Special Educational Needs. The asterisk (*) symbol is used as a wildcard, specifically for truncation.

**Table 2:** A summary table of the key features of the eight studies in the current systematic review.

Study (Authors, Year)	Population and Sample	Study Design and Methods	Focus and Measures	Key Findings and Implications
1. Salimi et al. (2023)	319 Iranian adults with visual impairments (considered within SEN contexts)	A cross-sectional survey design using standardized self-report measures	Examined the associations among career adaptivity, CA, and career satisfaction using validated scales; emphasized the interrelationship of these constructs in career development	Demonstrated a positive association among career adaptivity, CA, and career satisfaction, suggesting that integrating CA strategies into career training may enhance the career satisfaction in visually impaired individuals
2. Yang et al. (2023)	204 SEN students in Hong Kong	An instrument validation study using survey methodologies	Focused on validating the psychometric properties of the Career Adapt-Abilities Scale—Short Form (CAAS-SF); assessed the dimensional structure of CA and its relationship with self-esteem	Confirmed a robust four-factor structure for CA and identified a positive correlation between CA and self-esteem, supporting the scale’s utility for targeted career guidance initiatives in SEN populations
3. Yuen and Chan (2024)	345 SEN students and 237 matched participants in Hong Kong	A longitudinal study with two measurement points over an 18-month interval	Investigated predictors of CA and career self-efficacy by incorporating measures of meaning in life along with established CA scales; provided insight into internal motivational resources influencing vocational outcomes	Found that a strong sense of meaning in life significantly predicted both CA and career self-efficacy, underscoring the importance of internal psychological resources in driving career development among SEN students
4. Stevenson et al. (2021)	85 individuals with serious mental illnesses in the USA	A cross-sectional survey design utilizing self-report questionnaires	Explored the roles of work hope and CA in determining job search intensity; employed established measures for work hope, CA, and job search behaviors to elucidate their interplay in a clinical sample	Revealed that work hope significantly predicted job search intensity while an unexpected negative correlation emerged between CA and job search intensity, indicating the need for refined measurement and context-specific interventions for populations with mental health challenges
5. Antonelli et al. (2018)	26 legally blind college students paired with 26 mentors in the USA	An intervention study replicating a mentoring program design	Evaluated the effectiveness of a structured mentoring program on enhancing job search behaviors and CA; measured changes in CA using established scales before and after the intervention	Reported that the mentoring program led to significant improvements in efficient job searching and enhanced CA, thereby confirming the potential of mentoring as a practical intervention in SEN contexts
6. Tokar and Kaut (2018)	320 workers diagnosed with Chiari malformations in the USA	A cross-sectional study employing a mediation analysis with self-report instruments	Assessed the mediating role of CA in the inverse relationship between economic constraints and access to decent work; utilized measures to quantify economic constraints, CA, and quality of work conditions	Found that CA partially mediated the relationship between economic constraints and access to decent work, suggesting that CA functions as a buffering resource in mitigating adverse vocational outcomes amid economic challenges
7. O’Mally and Antonelli (2016)	26 legally blind college students paired with 26 employed mentors in the USA	An intervention study focused on a mentoring program, with pre- and post-intervention assessments	Investigated the impact of a mentoring intervention on job-seeking assertiveness, career self-efficacy, and CA; employed validated scales to capture changes in these career-related constructs	Demonstrated that structured mentoring effectively improved job-seeking assertiveness, career self-efficacy, and CA, thereby supporting the implementation of targeted career development programs in SEN contexts
8. Santilli et al. (2014)	120 Italian adults with intellectual disabilities	Cross-sectional survey design using self-report questionnaires	Explored the interrelationships among CA, hope, and life satisfaction; measured CA alongside constructs of hope and overall well-being to assess mediating effects	Indicated that hope partially mediated the relationship between CA and life satisfaction, highlighting the potential value of incorporating positive psychological constructs into vocational interventions

Note: CA = Career Adaptability, SEN = Special Educational Needs.

## Data Availability

No new data were created or analyzed in this study. Data sharing is not applicable to this article.

## Appendix A

**Table A1:** Demographic details of all 48 screened records.

Inclusion in This Systematic Review (Yes/No)	Reasons for Exclusion/Inclusion	Authors	Year	Title	Key Takeaway	Document Type
Yes	Students with SEN	Yang L., Sin K.F., Savickas M.L.	2023	Assessing factor structure and reliability of the career adaptability scale in students with special educational needs	Validated the Career Adapt-Abilities Scale among students with special educational needs in Hong Kong	Article
Yes	Workers with ID	Santilli S., Nota L., Ginevra M.C., Soresi S.	2014	Career adaptability, hope and life satisfaction in workers with intellectual disability	Tested a mediation model linking career adaptability, hope, and life satisfaction in workers with intellectual disabilities	Article
Yes	Students with VI	Antonelli, Karla; Steverson, Anne; O’Mally, Jami	2018	College graduates with visual impairments: A report on seeking and finding employment.	Assessed the effects of mentoring on the job-seeking outcomes among college students with legal blindness	Article
Yes	Individuals with MI	Stevenson B.J., Millner U.C., Satgunam S.A., Love R.	2021	Hope, adaptability, and job-search intensity among individuals living with serious mental illness	Examined how work hope and career adaptability predicted job search intensity in individuals with serious mental illness	Article
Yes	Workers with CM	Tokar D.M., Kaut K.P.	2018	Predictors of decent work among workers with Chiari malformation: An empirical test of the psychology of working theory	Tested the Psychology of Working Theory for workers with chronic health conditions, including career adaptability as a mediator	Article
Yes	Students with VI	O’Mally J., Antonelli K.	2016	The effect of career mentoring on employment outcomes for college students who are legally blind	Studied the impact of mentoring on the job-seeking self-efficacy and career adaptability among visually impaired college graduates	Article
Yes	Students with SEN	Yuen M., Chan R.T.H.	2024	The influence of social connectedness and meaning in life on career adaptability and career self-efficacy in students with special educational needs	Tested the effects of meaning in life and social connectedness on career adaptability and self-efficacy among SEN students	Article
Yes	Individuals with VI	Salimi S., Nilforooshan P., Sadeghi A.	2023	Towards Career Satisfaction by Career Adaptation Model Among Individuals With Visual Impairment	Tested the career adaptation model in individuals with visual impairments, highlighting career adaptability as a mediator	Article
No	Non-SEN (no participants with special educational needs)	Autin, K. L., Shelton, A. J., Diaz Tapia, W. A., Garcia, R. G., & Cadenas, G. A.	2021	Testing psychology of working theory among Spanish-speaking Latinx workers in the U.S.	Validated Psychology of Working Theory constructs and tested their relevance among Latinx workers	Article
No	No full text	Meacham H., Bartram T., Cavanagh J.	2020	Career Management: The Transition Process for Workers with Disability	Discussed the career transition support for disabled workers using optimal distinctiveness theory	Book Chapter
No	No full text	Lindstrom, L., Hirano, K., & Thomas, R.	2018	Career development for individuals with disabilities: Examining issues of equity, access and opportunity.	Applied a Systems Theory Framework to understanding the career development for people with disabilities	Book Chapter
No	Non-empirical design	Ferrari L., Sgaramella T.M., Santilli S., Di Maggio I.	2017	Career adaptability and career resilience: The roadmap to work inclusion for individuals experiencing disability	Reviewed career adaptability and career resilience for individuals with and without disabilities; not an empirical study	Book Chapter
No	Non-CA (not career adaptability research)	Lent, Robert W.	2013	Career-life preparedness: Revisiting career planning and adjustment in the new workplace.	Proposed a career – life preparedness framework integrating career adaptability and coping	Article
No	Non-CA	Zhang, Jiahong; Chen, Gaowei; Yuen, Mantak	2019	Validation of the Vocational Identity Status Assessment (VISA) using Chinese technical college students.	Validated the Chinese version of the Vocational Identity Status Assessment	Article
No	Non-CA	Restubog, Simon Lloyd D.; Deen, Catherine Midel; Decoste, Anthony; He, Yaqing	2021	From vocational scholars to social justice advocates: Challenges and opportunities for vocational psychology research on the vulnerable workforce	Called for broader research into overlooked populations of vulnerable workers	Article
No	Non-CA	Yuan, Wei; Xie, Zhengli; Dong, Ping; Yang, Yuqin	2023	Linking perceived social support to self-esteem and social integration among adolescents with visual impairment: A cross-lagged study	Examined the links between social support, self-esteem, and integration in visually impaired adolescents	Article
No	Non-CA and non-SEN	Autin, Kelsey L.; Herdt, Megan E.; Allan, Blake A.; Zhu, Lina; Abdullah, Mukadas; Garcia, Roberto G.	2022	Decent work among women workers: An intersectional approach.	Tested an intersectional model of decent work for women experiencing racism and sexism	Article
No	Non-CA and non-SEN	Wehmeyer M.L., Nota L., Soresi S., Shogren K.A., Morningstar M.E., Ferrari L., Sgaramella T.M., DiMaggio I.	2019	A Crisis in Career Development: Life Designing and Implications for Transition	Reviewed the evolution of the career development models in transition services	Article
No	Non-SEN	Xue, Yuxiu	2022	A theoretical review on the interplay of EFL/ESL teachers’ career adaptability, self-esteem, and social support	Reviewed self-esteem, social support, and career adaptability in second/foreign language teachers	Article
No	Non-SEN	Datu, Jesus Alfonso D.; Buenconsejo, Jet U.	2021	Academic engagement and achievement predict career adaptability.	Analyzed how academic engagement and achievement predict career adaptability in Filipino students	Article
No	Non-SEN	Kara A., Orum-Çattık E., Eryılmaz A.	2022	Adaptivity, adaptability, adapting response, and adaptation result: testing with structural equation modelling on pre-service teachers	Tested a career construction model for academic and life satisfaction in special education teacher candidates	Article
No	Non-SEN	Wei, Jingyi; Chan, Sow Hup Joanne; Autin, Kelsey	2022	Assessing perceived future decent work securement among Chinese impoverished college students.	Tested the Psychology of Working Theory among impoverished Chinese students and explored the role of work volition	Article
No	Non-SEN	Duffy, Ryan D.; Douglass, Richard P.; Autin, Kelsey L.	2015	Career adaptability and academic satisfaction: Examining work volition and self efficacy as mediators.	Explored the mediators between career adaptability and academic satisfaction in undergraduates	Article
No	Non-SEN	Ozdemir N.K., Koç M.	2023	Career adaptability of parents of children with autism spectrum disorder	Investigated the career adaptability of parents of children with autism spectrum disorder during COVID-19	Article
No	Non-SEN	Prescod, Diandra J.; Zeligman, Melissa	2018	Career adaptability of trauma survivors: The moderating role of posttraumatic growth.	Explored how trauma symptoms and posttraumatic growth predict career adaptability in trauma survivors	Article
No	Non-SEN	Santilli, Sara; Grossen, Silke; Nota, Laura	2020	Career Adaptability, Resilience, and Life Satisfaction Among Italian and Belgian Middle School Students	Analyzed career adaptability and resilience as predictors of life satisfaction in middle school students in Belgium and Italy	Article
No	Non-SEN	Ginevra M.C., Di Maggio I., Santilli S., Sgaramella T.M., Nota L., Soresi S.	2018	Career adaptability, resilience, and life satisfaction: A mediational analysis in a sample of parents of children with mild intellectual disability†	Studied career adaptability and resilience as predictors of life satisfaction in parents of children with intellectual disabilities	Article
No	Non-SEN	Park, Soeun; Garrison, Yunkyoung Loh; Liu, William Ming	2020	Career decision ambiguity tolerance of Asian men in the United States.	Examined how career decision ambiguity tolerance influenced career behaviors and well-being in Asian men in the U.S.	Article
No	Non-SEN	PONG, Hok-Ko; LEUNG, Chi Hung	2023	Cross-sectional study of the relationship between trait emotional intelligence and career adaptability of Chinese youths	Investigated how trait emotional intelligence predicted career adaptability among Chinese university students in Hong Kong	Article
No	Non-SEN	Duffy R.D., Gensmer N., Allan B.A., Kim H.J., Douglass R.P., England J.W., Autin K.L., Blustein D.L.	2019	Developing, validating, and testing improved measures within the Psychology of Working Theory	Developed new measures of marginalization and economic constraints and tested the Psychology of Working Theory using career adaptability	Article
No	Non-SEN	Lee, Ji Hae; Cho, Soohyun; Lee, Sujung; Eum, Wonsun Jini; Jang, Hansori; Suh, Suhyun; Lee, Sang Min	2017	Initial validation of the Planned Happenstance Career Inventory–English version.	Validated the cross-cultural structure of the Planned Happenstance Career Inventory in U.S. students	Article
No	Non-SEN	Ma, Yin; Huang, Genghua; Autin, Kelsey L.	2021	Linking decent work with academic engagement and satisfaction among first-generation college students: A psychology of working perspective.	Tested a mediation model where career adaptability and work volition predicted academic outcomes based on Psychology of Working Theory	Article
No	Non-SEN	Ulrike Fasbender, Anne Burmeister, Mo Wang	2020	Motivated to be socially mindful: Explaining age differences in the effect of employees’ contact quality with coworkers on their coworker support	Analyzed how coworker contact and age influenced support behaviors using social mindfulness and socioemotional selectivity theories	Article
No	Non-SEN	Richard P. Douglass, Kelsey L. Autin, Aysenur Buyukgoze-Kavas, and Nicholas P. Gensmer	2020	Proactive personality and decent work among racially and ethnically diverse working adults.	Tested Psychology of Working Theory predictors of decent work, including career adaptability, in a diverse adult sample	Article
No	Non-SEN	Yuen, Mantak; Yau, Josephine	2015	Relation of career adaptability to meaning in life and connectedness among adolescents in Hong Kong.	Explored how meaning in life and school connectedness predicted career adaptability components in Chinese adolescents	Article
No	Non-SEN	Autin, Kelsey L.; Douglass, Richard P.; Duffy, Ryan D.; England, Jessica W.; Allan, Blake A.	2017	Subjective social status, work volition, and career adaptability: A longitudinal study.	Examined the longitudinal relations between social status, work volition, and career adaptability in undergraduates	Article
No	Non-SEN	Leung, S. Alvin; Mo, Jianhong; Yuen, Mantak; Cheung, Raysen	2022	Testing the career adaptability model with senior high school students in Hong Kong.	Tested the career adaptability model from career construction theory in Hong Kong high school students	Article
No	Non-SEN	Huebner R.A., Emery L.J., Shordike A.	2002	The adolescent role assessment: Psychometric properties and theoretical usefulness	Assessed the utility of the Adolescent Role Assessment in measuring career adaptability among adolescents	Article
No	Non-SEN	Hyung Joon Yoon, Natasha Bailey, Norman Amundson, and Spencer Niles	2019	The effect of a career development programme based on the Hope-Action Theory: Hope to work for refugees in British Columbia.	Evaluated the effects of a Hope–Action Theory-based training on career adaptability in refugees	Article
No	Non-SEN	Er, Bay Yan; Mohd Rameli, Mohd Rustam	2019	The influence of emotional intelligence and personality on career adaptability among teachers in special education schools in Johor Bahru	Investigated how emotional intelligence and personality traits predicted career adaptability in Malaysian special education teachers	Article
No	Non-SEN	Bettonville, Brian P.	2018	The Psychology of Working Theory: Predicting decent work experiences.	Tested the Psychology of Working Theory among working adults by linking marginalization, economic resources, and career adaptability	Dissertation
No	Non-SEN	Sou, Elvo K. L.; Yuen, Mantak; Chen, Gaowei	2022	Career adaptability as a mediator between social capital and career engagement.	Validated the Chinese short form of the Career Adapt-Abilities Scale and tested its mediation role between social capital and career engagement	Article
No	Non-SEN	Duffy, Ryan D.; Velez, Brandon L.; England, Jessica W.; Autin, Kelsey L.; Douglass, Richard P.; Allan, Blake A.; Blustein, David L.	2018	An examination of the Psychology of Working Theory with racially and ethnically diverse employed adults	Found that greater economic resources and lower marginalization led to increased work volition, which in turn enhanced the career adaptability and access to decent work, among racially and ethnically diverse employed adults	Article
No	Non-SEN	Klehe U.-C., Fasbender U., van der Horst A.	2021	Going full circle: Integrating research on career adaptation and proactivity	Integrated career adaptability and career proactivity literatures into a common framework using the Rubicon model	Article
No	Non-SEN	Agoes Salim, Rose Mini; Istiasih, Mirna Refolia; Rumalutur, Nur Aisyah; Biondi Situmorang, Dominikus David	2023	The role of career decision self-efficacy as a mediator of peer support on students’ career adaptability	Tested career decision self-efficacy as a mediator between peer support and career adaptability among Indonesian college students	Article
No	Study protocol	Simmons M.B., Nicholas J., Chinnery G., O’Sullivan S., D’Alfonso S., Bendall S., Cagliarini D., Hamilton M., Gleeson J., Killackey E., Alvarez-Jimenez M.	2021	The youth online training and employment system: Study protocol for a randomized controlled trial of an online vocational intervention for young people with mental ill health	Evaluated an online intervention aiming to enhance the career adaptability among youth with mental ill health	Article
No	Non-SEN	Kim H.J., Kim S.Y., Duffy R.D., Nguyen N.P., Wang D.	2020	A cross-cultural comparison of psychology of working theory among U.S. and Korean college students	Tested a modified Psychology of Working Theory model with US and Korean students including career adaptability	Article
No	Non-SEN	Kim H.J., Duffy R.D., Lee S., Lee J., Lee K.-H.	2019	Application of the Psychology of Working Theory With Korean Emerging Adults	Examined career adaptability within Psychology of Working Theory among Korean emerging adults	Article

Note: non-SEN = no participants with special educational needs; non-CA = not career adaptability research; ID = intellectual disability; VI = visual impairment; MI = mental illness; CM = Chiari malformation.

**Table A2:** The PRISMA 2020 checklist for the current systematic review.

Checklist Item	Location in Manuscript/Notes
1. Title	Identified as a systematic review in the title.
2. Abstract	A structured abstract following the PRISMA 2020 abstract guidelines.
3. Rationale	The introduction establishes the gap in our understanding of the career adaptability among SEN populations.
4. Objectives	The review aims to synthesize the literature on career adaptability in SEN students.
5. Eligibility criteria	Detailed inclusion and exclusion criteria described in Methods.
6. Information sources	Searched five databases: Web of Science, PsycINFO, ProQuest, Scopus, and PubMed.
7. Search strategy	The full search strategy and rationale are presented in Table 1.
8. Selection process	The PRISMA 2020 flow diagram outlines the multi-phase screening process.
9. Data collection process	Data extraction summarized; full demographic and study characteristics given in Table 2 and Appendix A (Table A1).
10a. Data items (outcomes)	Career adaptability constructs were examined across the included studies.
10b. Data items (other variables)	Demographic and contextual factors were extracted and summarized.
11. Study risk of bias assessment	Not assessed due to the scope and the qualitative nature of synthesis.
12. Effect measures	Narrative synthesis only; no quantitative effect sizes reported.
13a. Synthesis eligibility	Only studies matching the inclusion criteria were included after the eligibility review.
13b. Data preparation	Not applicable—no data transformation required.
13c. Tabulation or display	The study data are summarized in the tables and Appendix A.
13d. Synthesis methods	Narrative synthesis structured around key themes.
13e. Heterogeneity exploration	Not applicable—no quantitative synthesis.
13f. Sensitivity analysis	Not applicable. A sensitivity analysis was not conducted because the review involved a qualitative narrative synthesis, not a quantitative meta-analysis where such procedures are typically used to assess the robustness of the statistical findings under alternative assumptions. Since no statistical models or pooled estimates were applied, there were no analytic decisions requiring sensitivity testing, such as the exclusion of high-risk studies or varying inclusion criteria.
14. Reporting bias assessment	Not conducted due to scope. An assessment of reporting bias (e.g., publication bias or selective outcome reporting) was not conducted because the review’s scope was descriptive and exploratory, focusing on mapping the existing literature on career adaptability in SEN populations. Tools for detecting reporting biases rely on quantitative synthesis and homogeneity across studies, which were not applicable in this context.
15. Certainty assessment	Not assessed due to qualitative synthesis. The certainty or confidence in the body of evidence (e.g., using the Grading of Recommendations Assessment, Development and Evaluation (GRADE) system, Guyatt et al., 2008) was not assessed because the review adopted a qualitative synthesis approach without quantitative effect estimates.
16a. Study selection results	The flow of studies is clearly presented in the PRISMA 2020 diagram (Figure 1).
16b. Excluded studies	The reasons for exclusion are detailed in the main text and diagram (see Figure 1).
17. Study characteristics	Details in the main text and Appendix A.
18. Risk of bias in studies	Not assessed due to the qualitative and scoping nature of the synthesis. The review aimed to map the landscape of the existing research rather than to evaluate the methodological rigor or compare the effect sizes across studies. This aligns with the accepted practice in narrative or scoping reviews.
19. Results of individual studies	Narrative summaries provided for each included study.
20a–d. Results of syntheses	Narrative synthesis structured thematically; no quantitative analysis.
21. Reporting biases	Not assessed. Reporting bias (e.g., publication bias or selective outcome reporting) was not formally assessed because the review adopted a qualitative synthesis approach without statistical pooling. The standard methods for detecting reporting biases (e.g., funnel plots, Egger’s test) require effect sizes and variance estimates across multiple studies, which were not applicable given the narrative and heterogeneously scoped evidence base.
22. Certainty of evidence	Not applicable. The certainty of evidence (e.g., using GRADE) was not assessed because the review did not aim to evaluate the effect of interventions or make strength-of-evidence claims. The review was exploratory in nature, seeking to synthesize the scope and characteristics of and conceptual trends in the literature on career adaptability in SEN contexts. The diversity of the studies’ designs, aims, and measures precluded the meaningful application of quantitative certainty grading systems. In line with PRISMA guidance, certainty assessments are only required when a review draws on quantitative effect estimates to inform practice or policy, which was not the objective here.
23. Discussion	Findings interpreted contextually with reference to limitations and implications.
24a–c. Registration and protocol	The review was not registered, and no prior protocol was cited.
25. Support	Institutional or funding support acknowledged in Acknowledgements.
26. Competing interests	The authors declared no competing interests.
27. Availability of data and materials	Appendix A includes all of the screened and included studies, extracted data tables, reasons for exclusion, and the PRISMA checklist. Additional materials are available upon request.

Note: SEN = Special Educational Needs; PRISMA = Preferred Reporting Items for Systematic reviews and Meta-Analyses; GRADE = Grading of Recommendations Assessment, Development and Evaluation.

**Table A3:** Descriptive alternative text for Figure 2 on CCT and career adaptability research.

Element	Description
Figure Title	Integrating and strengthening Career Construction Theory (CCT) as a solid theoretical framework for future empirical research on career adaptability (CA) among individuals with special educational needs (SEN): Career Construction Theory (CCT) offers a holistic approach to understanding careers, focusing on the interplay between individual narratives, adaptability, and social context.
Figure Layout	A two-column structure with a curved two-way arrow connecting the left (current research) and the right (future research). The left column is in blue, and the right column is in orange.
Left Column	**Current systematic review on CA** (Examined and observed in eight empirical studies among individuals with SEN): • Positive links between CA and career outcomes • Mentoring as an intervention strategy • Validation of CA measures for SEN students • Meaning in life as a motivational predictor • CA as a mediator against adversity • Mixed findings in mental health contexts • Hope as a mechanism linking CA to well-being • Limited age and disability group diversity among the eight studies • Geographical and contextual concentration on high-income countries and regions (e.g., USA, Hong Kong, Italy) • Cross-sectional dominance with emerging longitudinal efforts
Right Column	**Future research on CA** (Key aspects to be examined in future empirical research on CA among individuals with SEN): • Mapping career adaptability development in early and middle adolescence through career construction processes • Advancing career construction research among SEN and neurodiverse individuals • Extending career construction theory across cross-cultural/socioeconomic contexts • Modeling dynamic trajectories of career adaptability aligned with CCT • Investigating contextual/ecological moderators shaping career construction and adaptation • Integrating emotion regulation within career construction’s adaptive readiness framework • Designing differentiated and accessible career adaptability measures for SEN populations grounded in career construction theory • Innovating school-to-work transition interventions for SEN individuals through career-construction-based approaches • Employing multi-informant and multi-method designs to capture the complexity of career construction in SEN contexts • Linking early career adaptability to long-term career and life design outcomes within a career construction perspective, etc.
Theoretical Message	Career construction theory offers a holistic approach to understanding careers, emphasizing the interplay between personal narratives, adaptability, and social context.
Accessibility Enhancements	The figure was redesigned in SVG format using Calibri font, high-contrast colors, ample white space, and descriptive labels to ensure screen reader and accessibility compliance.

Notes: CCT = Career Construction Theory; CA = Career Adaptability; SEN = Special Educational Needs.
